# *Wolbachia* strain *w*AlbB maintains high density and dengue inhibition following introduction into a field population of *Aedes aegypti*

**DOI:** 10.1098/rstb.2019.0809

**Published:** 2020-12-28

**Authors:** Noor Afizah Ahmad, Maria-Vittoria Mancini, Thomas H. Ant, Julien Martinez, Ghazali M. R. Kamarul, Wasi A. Nazni, Ary A. Hoffmann, Steven P. Sinkins

**Affiliations:** 1 Institute for Medical Research, Ministry of Health Malaysia, Jalan Pahang, 50588 Kuala Lumpur, Malaysia; 2 MRC-University of Glasgow Centre for Virus Research, University of Glasgow, 464 Bearsden Road, Glasgow G61 1QH, UK; 3 University of Melbourne, Bio21 Institute and the School of BioSciences, 30 Flemington Road, Parkville, Victoria 3052, Australia

**Keywords:** *Wolbachia*, *Aedes aegypti*, *w*AlbB, dengue virus, vector control, virus blocking

## Abstract

*Aedes aegypti* mosquitoes carrying the *w*AlbB *Wolbachia* strain show a reduced capacity to transmit dengue virus. *w*AlbB has been introduced into wild *Ae. aegypti* populations in several field sites in Kuala Lumpur, Malaysia, where it has persisted at high frequency for more than 2 years and significantly reduced dengue incidence. Although these encouraging results indicate that *w*AlbB releases can be an effective dengue control strategy, the long-term success depends on *w*AlbB maintaining high population frequencies and virus transmission inhibition, and both could be compromised by *Wolbachia–*host coevolution in the field. Here, *w*AlbB-carrying *Ae. aegypti* collected from the field 20 months after the cessation of releases showed no reduction in *Wolbachia* density or tissue distribution changes compared to a *w*AlbB laboratory colony. The *w*AlbB strain continued to induce complete unidirectional cytoplasmic incompatibility, showed perfect maternal transmission under laboratory conditions, and retained its capacity to inhibit dengue. Additionally, a field-collected *w*AlbB line was challenged with Malaysian dengue patient blood, and showed significant blocking of virus dissemination to the salivary glands. These results indicate that *w*AlbB continues to inhibit currently circulating strains of dengue in field populations of *Ae. aegypti*, and provides additional support for the continued scale-up of *Wolbachia* wAlbB releases for dengue control.

This article is part of the theme issue ‘Novel control strategies for mosquito-borne diseases’.

## 1. Background

Releases of *Aedes aegypti* carrying the maternally inherited bacterial endosymbiont *Wolbachia* are being trialled in several countries as a novel arbovirus intervention [1–3]. Certain strains of *Wolbachia* possess attributes ideal for vector control; they have a capacity to invade mosquito populations while simultaneously reducing the transmission potential for important arboviruses, including dengue. The capacity of *Wolbachia* to spread through a population arises from a mating incompatibility (commonly known as cytoplasmic incompatibility, CI) between *Wolbachia*-carrying males and *Wolbachia*-free females, which renders the resulting progeny inviable, while *Wolbachia*-carrying females are able to reproduce successfully with both *Wolbachia*-carrying and *Wolbachia*-free males. *Ae. aegypti* carrying the *w*AlbB *Wolbachia* strain have been released in several sites in Kuala Lumpur, Malaysia, resulting in stable establishment in some areas. Residents of sites with high *w*AlbB frequencies experienced a decline in dengue incidence of approximately 40% compared to controls, although this is likely an underestimate of the true decrease in transmission given the potential for exposure outside of the release areas [1].

Levels of viral inhibition vary substantially between *Wolbachia* strains, ranging from no inhibition to complete blocking [4–6]. Different strains replicate to different levels within a host, and with a few exceptions, the level of viral blocking generally shows a positive correlation with *Wolbachia* intracellular density [6,7]. The distribution of *Wolbachia* in host tissues is also important for transmission blocking given evidence that viral inhibition is cell autonomous [8,9]. To achieve transmission in a mosquito, an arbovirus present in a bloodmeal must invade the midgut epithelium, disseminate in the haemolymph, and eventually establish an infection in the salivary glands. The presence of *Wolbachia* in the somatic tissues of the midgut and salivary glands is, therefore, central to the transmission-blocking phenotype. The high-density strains *w*MelPop and *w*Au reach very high somatic densities and generate particularly strong transmission blocking [4,8], although high *Wolbachia* densities are also associated with virulence in the host, negatively affecting a range of life-history traits including fecundity, longevity and egg survival over extended periods of quiescence [4,10]. Higher density strains, therefore, have a lower invasion potential which can limit their use in field interventions. *w*MelPop-carrying *Ae. aegypti* were released in field sites in Australia and Vietnam and despite reaching high initial frequencies, the strain was lost once releases ceased [11]. *w*AlbB reaches intermediate densities in *Ae. aegypti* and has a relatively low impact on many aspects of host fitness [4,12], while providing significant inhibition of dengue transmission [4,13,14].

A variety of environmental and symbiont/host genetic factors influence the density and tissue distribution of *Wolbachia*. High larval breeding site temperatures, for example, cause dramatic reductions in the density of some strains [4,15,16], although *w*AlbB appears to be relatively heat stable [4,16]. Host factors are important in restricting *Wolbachia* tropism to the germline in native associations; *w*AlbB is largely restricted to the germline in its native host *Aedes albopictus* [17], whereas *w*AlbB in *Ae. aegypti* has a broad somatic distribution [4]. Interestingly, transfer of the *w*Mel strain (native to *Drosophila melanogaster*) into *Ae. albopictus* results in high densities in midgut and salivary gland tissue [17] and strong viral inhibition [18]; thus restricted tissue tropism within a particular host species can be *Wolbachia* strain-specific. In addition to inter-species variation, intra-species differences may also play an important role. Experimental evolution of *w*Mel-carrying *Ae. aegypti* generated differences in virus inhibition among *w*Mel-carrying lineages which correlated with genetic changes in the host genome, although this was independent of *Wolbachia* density [19].

As with any vector control method, evolutionary responses have the potential to disrupt the long-term stability of a *Wolbachia*-based intervention. Since *Wolbachia* is maternally transmitted, the symbiosis is expected to evolve towards a more benign or even mutualistic association through adaptations of the host, the symbiont or both [20]. Given that virus transmission blocking is largely governed by *Wolbachia* densities in somatic tissues, and that high titre somatic *Wolbachia* can be virulent, natural selection may act to decrease densities in the midgut and salivary glands, leading to a reduction in overall viral inhibition. However, an eventual loss of viral inhibition is not necessarily the default evolutionary outcome. Virus inhibition may even be selected for; a recent study reported that a lineage of *w*Mel-carrying *Ae. aegypti* that exhibited lower virus inhibition also had reduced relative fitness [19]. Importantly, the authors of this study managed to select for lower virus inhibition from field-collected mosquitoes through experimental evolution, while failing to select for stronger inhibition, indicating that lineages with strong inhibition persist at high frequency in the field. Furthermore, *Wolbachia*-mediated resistance to pathogenic insect-specific viruses may act as a source of selection to maintain the virus inhibition phenotype, although the magnitude of this selective pressure remains to be determined in wild mosquito populations. Consistent with a lack of strong selection on *Wolbachia* or host, the *w*Mel strain has maintained both virus inhibition and deleterious fitness traits in Australian populations of *Ae. aegypti* nearly a decade after introduction [21,22].

Given the potential for host genotype effects on viral inhibition, and the possibility of host and symbiont evolution, long-term monitoring of field populations for phenotypic stability forms an important part of the routine surveillance of a *Wolbachia* intervention. The *w*AlbB-carrying *Ae. aegypti* strain used in field releases in Malaysia was originally transferred into an *Ae. aegypti* colony isolated from wild-caught mosquitoes in Kuala Lumpur in the 1960s, and showed complete CI induction, 100% maternal transmission and inhibition of dengue transmission [4]. After backcrossing to field-collected males to improve field performance particularly within the context of pesticide resistance, the *w*AlbB strain was released in a number of sites in urban Kuala Lumpur, including Mentari Court (a set of seven 18-floor apartment buildings), and Shah Alam (§7) a commercial/residential zone. Releases in Mentari Court ceased on the 5 March 2018, with *w*AlbB spreading rapidly and maintaining greater than 90% frequency 2 years later [1]. Releases in Shah Alam ceased on 20 April 2019, with *w*AlbB maintaining a frequency of greater than 84% more than a year later. Given the far greater genetic diversity of wild compared to laboratory populations, and a higher degree of mating competition and selection on mosquito fitness in the field, there may be faster evolution towards lower *w*AlbB densities in the field versus the laboratory. To assess whether the field population has undergone an accelerated rate of selection on density we examined a colony of *w*AlbB-carrying *Ae. aegypti* recently established from mosquitoes collected in Mentari Court, 20 months post cessation of field releases. *Wolbachia* density and tissue distribution, the stability of the DENV-2 dengue blocking phenotype, CI and maternal transmission rates are assessed and compared to a laboratory *w*AlbB colony. In addition to evaluating the post-release phenotypic stability of *w*AlbB, the competence of a *w*AlbB field strain established from the Shah Alam release site was evaluated through oral challenge with DENV-1 infected blood from a Malaysian dengue patient.

## 2. Results

### (a) *Wolbachia* density and tissue distribution

**Figure 1.**
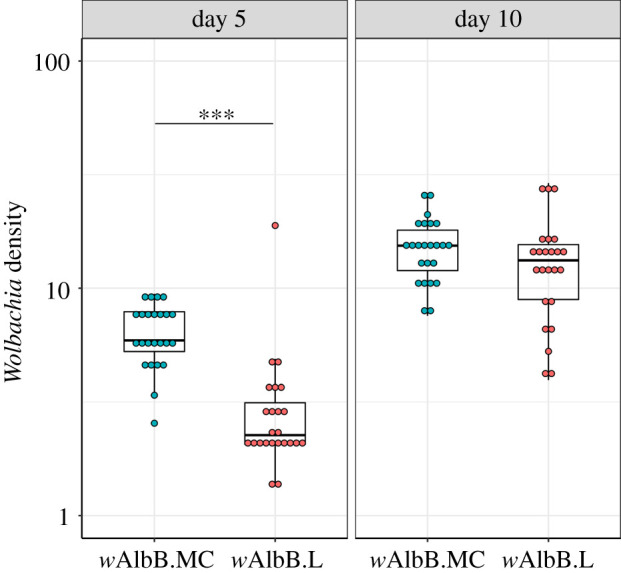
*Wolbachia* density in whole adult females. *Wolbachia* density was determined at 5 and 10 days post adult eclosion by qPCR. Twenty-four females were analysed per group. Individual dots represent *Wolbachia* densities in individual females. A significant difference was found between *w*AlbB.MC and *w*AlbB.L densities at day 5 (pairwise *t-*test, *p* < 0.0001), although no difference was found at day 10 (*p* = 0.17). Boxplots show median and interquartile range.

The densities of *Wolbachia* in whole adult females reared from the F_3_ eggs of a *w*AlbB-carrying *Ae. aegypti* line established from the Mentari Court release site in Kuala Lumpur (hereon *w*AlbB.MC) were measured at 5 and 10 days post-eclosion (PE) and compared to densities in the laboratory *w*AlbB colony (hereon *w*AlbB.L). *w*AlbB density increased with age in both mosquito lines (figure 1). The whole-body *Wolbachia* density in the *w*AlbB.MC line was found to be significantly higher than that in the *w*AlbB.L line at day-5 PE, although densities between the lines at day-10 PE were similar.

**Figure 2.**
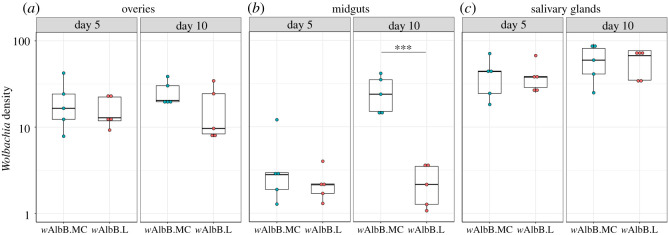
*Wolbachia* density in dissected tissues across mosquito lines and ages. Ovaries, midguts and salivary glands were dissected at days 5 and 10 post adult eclosion, and *w*AlbB density was determined by qPCR. Each of the five dots represents the *Wolbachia* density from pools of tissue from three individual female mosquitoes. No significant differences were observed in ovary density (line effect: *F*_1,16_ = 0.97, *p* = 0.33; age effect: *F*_1,16_ = 0.02, *p* = 0.89; line-by-age interaction: *F*_1,16_ = 0.55, *p* = 0.47) or salivary gland density (line effect: *F*_1,16_ = 0.004, *p* = 0.95; age effect: *F*_1,16_ = 3.39, *p* = 0.08; line-by-age interaction: *F*_1,16_ = 0.002, *p* = 0.96). A significantly higher *Wolbachia* density was observed in the midguts of the *w*AlbB.MC line at day 10 (line effect: *F*_1,16_ = 26.77, *p* < 0.0001; age effect: *F*_1,16_ = 14.45, *p* = 0.0016; line-by-age interaction: *F*_1,16_ = 14.96, *p* = 0.0014), but not at day 5 (pairwise *t-*test, *p* = 0.6). Boxplots show median and interquartile range. Five biological replicates were performed for each treatment.

Densities were also measured in the dissected ovary, midgut and salivary gland tissues from F_4_ females. *w*AlbB density in the ovaries was stable over time and between mosquito lines with no statistical differences between the groups (figure 2*a*). In midguts, densities differed between ages and mosquito lines (figure 2*b*). While at day-5 PE, there was no difference in midgut *w*AlbB levels between the two lines, at day-10 PE the *Wolbachia* density was significantly higher in the *w*AlbB.MC line compared to *w*AlbB.L. *Wolbachia* density in salivary glands was similar between mosquito lines at all ages (figure 2*c*).

### (b) Maternal transmission and cytoplasmic incompatibility

Rates of *Wolbachia* maternal transmission in the *w*AlbB.MC line were assessed in the progeny of crosses between wild-type (*Wolbachia-*free) males and *w*AlbB.MC females (i.e. in the absence of CI). All the G_1_ offspring were found to carry *w*AlbB out of 48 progeny assessed at the larval stage, indicating high rates of maternal transmission (binomial confidence intervals, 92.6–100%).

Reciprocal crosses between the *w*AlbB.MC line and the *Wolbachia*-free wild-type line indicated the retention of full uni-directional CI, with no eggs hatching from crosses between *w*AlbB.MC males and wild-type females (table 1). No significant difference in hatch rate was observed between *w*AlbB.MC males and females, and wild-type males and *w*AlbB.MC females, indicating no loss in capacity to rescue CI.

**Table 1.** Hatch rates from reciprocal crosses between *w*AlbB.MC and wild-type *Ae. aegypti*. Percentages represent egg hatch rates and the total number of eggs assessed is shown in parentheses. No difference in hatch rate was observed between crosses of *w*AlbB.MC males and females, and wild-type males and *w*AlbB.MC females (*p* > 0.8, Fisher's Exact test)*.*

		male	
		wild type	*w*AlbB.MC
female	wild type	67% (311)	0% (181)
	*w*AlbB.MC	63% (205)	63% (424)

### (c) Dengue inhibition

**Figure 3.**
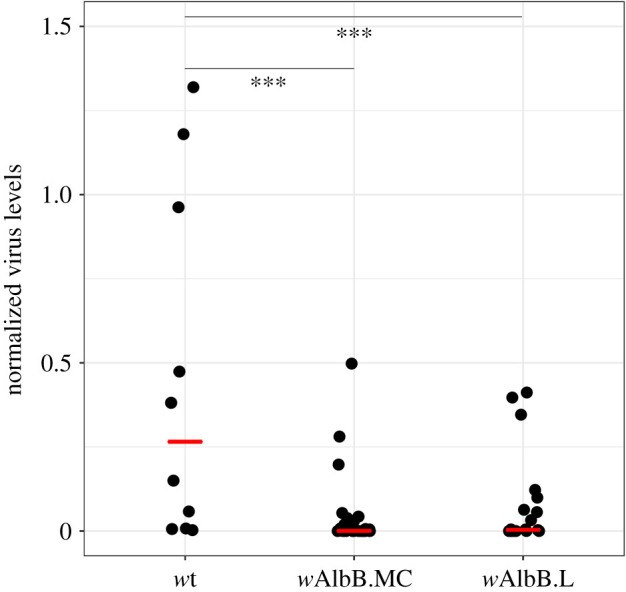
DENV-2 inhibition in *w*AlbB-carrying *Ae. aegypti* lines. Females from the *w*AlbB.MC, *w*AlbB.L and wild-type lines were fed a bloodmeal spiked with DENV-2. After an incubation period of 12 days, salivary glands were dissected and the viral load was quantified by RT-qPCR. Ten, 20 and 27 females were analysed for the wild-type, *w*AlbB.L and *w*AlbB.MC groups, respectively. Black dots indicate salivary glands from individual mosquitoes. Red lines indicate median values. *w*AlbB.MC and *w*AlbB.L showed a significant reduction in viral titres (*p* < 0.004 for both comparisons, one-way ANOVA with Dunnett's). There was no difference in viral load between the *w*AlbB.MC and *w*AlbB.L lines (*p* = 0.88, one-way ANOVA with Dunnett's).

To determine the dengue inhibition capacity of the *w*AlbB.MC line, females from the *w*AlbB.MC and *w*AlbB.L lines, and a *Wolbachia-*free wild-type strain recently established from field-caught mosquitoes from Kuala Lumpur were orally challenged with a DENV-2-spiked bloodmeal. Viral load in dissected salivary gland tissues was assessed by qRT-PCR 12 days post-challenge. Both the *w*AlbB.MC and *w*AlbB.L lines showed a large and significant reduction in the levels of virus in salivary glands compared to the wild-type control (figure 3). Although the *w*AlbB.MC line showed a lower mean viral load than the *w*AlbB.L line, the difference between the *w*AlbB-carrying lines was not statistically significant.

**Figure 4.**
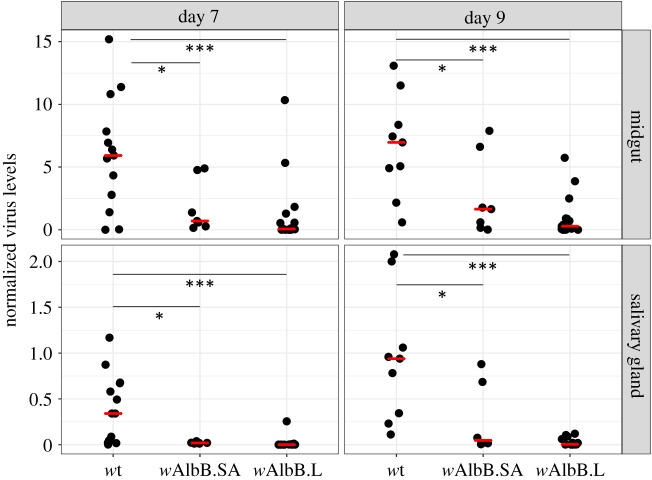
DENV-1 inhibition in *w*AlbB lines challenged with patient-derived blood. Females from the *w*AlbB.SA, *w*AlbB.L and wild-type lines were fed on clinical isolates of DENV-1 infected patient blood. After incubation periods of 7 and 9 days, midgut and salivary gland tissues were dissected, and the viral load was quantified by RT-qPCR. For midguts, tissues from 7, 18 and 13 females were analysed at day 7, and tissues from 7, 15 and 9 females were analysed at day 9 for the *w*AlbB.SA, *w*AlbB.L and wild-type groups, respectively. For salivary glands, tissues from 7, 16 and 13 females were analysed at day 7, and tissues from 6, 14 and 9 females were analysed at day 9 for the *w*AlbB.SA, *w*AlbB.L and wild-type groups, respectively. Black dots indicate tissues from individual mosquitoes. Red lines indicate median values. For midgut tissue, viral RNA titres in *w*AlbB.SA and *w*AlbB.L were significantly different from wild-type at day 7 (*p* < 0.004 for both *w*AlbB.SA and *w*AlbB.L, one-way ANOVA with Dunnett's) and at day 9 (*p* < 0.002 for *w*AlbB.SA *w*AlbB.L, one-way ANOVA with Dunnett's). Similarly, for salivary gland tissue, viral RNA titres in *w*AlbB.SA and *w*AlbB.L were significantly different from wild-type at day 7 (*p* < 0.0001 for both *w*AlbB.SA and *w*AlbB.L, one-way ANOVA with Dunnett's) and at day 9 (*p* < 0.0002 for *w*AlbB.SA *w*AlbB.L, one-way ANOVA with Dunnett's).

A second *w*AlbB-carrying field-derived line was established from mosquito larvae collected at the Shah Alam release site in Kuala Lumpur (hereon *w*AlbB.SA). To assess the capacity of *w*AlbB to block isolates of dengue virus currently circulating in Malaysia, females from the *w*AlbB.SA, *w*AlbB.L and wild-type lines were challenged with blood from DENV-1 infected patients. Individual female mosquitoes were dissected at 7 and 9 days post oral challenge, and midgut and salivary gland tissues were assessed for the abundance of viral RNA. Statistically significant reductions in the levels of DENV-1 RNA were detected in both the midgut and salivary gland tissues of both the *w*AlbB.SA and *w*AlbB.L lines relative to the *Wolbachia*-uninfected wild-type strain (figure 4). No significant differences in *Wolbachia* density were observed between the dengue challenged adults from the *w*AlbB.SA and *w*AlbB.L lines (electronic supplementary material, figure S1).

## 3. Discussion

*w*AlbB-carrying *Ae. aegypti* collected from a field site in Kuala Lumpur 20 months following the cessation of releases [1] did not show significant reductions in overall *Wolbachia* density or a diminished somatic density and tissue distribution compared to a laboratory colony [4], suggesting an absence of strong selection in the field. In fact, densities were found to be higher in the field line in whole bodies and midgut tissues for some time points. Further work will be needed to determine whether this difference is maintained with broader sampling and whether it is driven by host and/or symbiont genetic factors, or other variables such as the microbiota. Importantly, *w*AlbB in the field line continues to effectively inhibit the capacity for dengue virus to disseminate to and infect the salivary glands of females, thereby reducing transmission potential, with levels of viral inhibition similar to those observed in the laboratory *w*AlbB line and comparable to that observed in similar challenges performed in the ancestral line soon after generation [4]. Additionally, a *w*AlbB-carrying field-derived line strongly inhibited dissemination of dengue virus to the salivary glands when challenged with clinical isolates of DENV-1 infected blood recently collected from a hospitalized patient in Kuala Lumpur. These results indicate at least a medium-term stability of *w*AlbB density and dengue blocking in field populations of *Ae. aegypti*. The results from the clinical blood challenges are particularly encouraging given that estimates of virus blocking are often lower when patient blood is used compared to laboratory-prepared viremic bloodmeals [23,24], and that the clinical isolate was infected with DENV-1, a serotype that has previously shown lower susceptibility to *Wolbachia*-mediated inhibition relative to other serotypes [13,24].

As high *Wolbachia* densities in somatic tissues are correlated with reduced fitness, selection may be expected to favour the evolution of genetic factors capable of limiting symbiont tropism to those tissues essential for maternal transmission and CI, i.e. the germline. While somatic *Wolbachia* do occur in many co-evolved native *Wolbachia–*host combinations [25], in some native associations, such as those found in *Ae. albopictus* [17] and *Glossina morsitans* [26], *Wolbachia* is largely restricted to the ovaries and testes of the host. Somatic tissue distribution following transfer into *Ae. aegypti* tends to be broad, with particularly high densities in salivary gland tissues [4]. The rate of evolution of host or *Wolbachia* factors that limit somatic densities is likely to depend on the fitness costs associated with the symbiosis, with higher costs resulting in stronger and more rapid selection for restricted tropism. Artificial transfer of the virulent *w*MelPop *Wolbachia* strain to *Drosophila simulans*, for example, resulted in high initial fitness costs through reductions in fecundity and egg hatch rates, which were partially attenuated after several generations [27]. This was accompanied by reductions in density, and suggests rapid selection for host and/or symbiont genotypes capable of suppressing *Wolbachia* over-replication. By contrast, however, severe fitness costs caused by *w*MelPop have persisted for over a decade in a transinfected line of *Ae. aegypti*, suggesting stability of the deleterious phenotypes in this species [28]. Densities of *w*AlbB in the Malaysian field population were found to be similar to those of the laboratory colony 20 months after introduction, with densities remaining high in the somatic tissues of the midgut and salivary glands. Similar to measurements made in the ancestral *w*AlbB line performed shortly after strain generation [4], *Wolbachia* densities in the salivary glands of the field *w*AlbB population were several-fold those in the ovaries. Although *w*AlbB does cause a small reduction in adult longevity in laboratory-reared *Ae. aegypti*, the biological relevance to the field populations is uncertain given the average lifespan of wild mosquitoes is expected to be much shorter [29]. Furthermore, selection for reduced *w*MelPop densities in *D. simulans* may be particularly rapid as it is a native *Wolbachia* host; existing host factors capable of suppressing native strains may be active against novel associations, although these factors may require some adaptative optimization against divergent *Wolbachia* strains. When *w*AlbB was transferred into *Culex quinquefasciatus*, for example, it showed a limited somatic tissue distribution similar to the native *w*Pip strain—with which it is closely related—while the more distantly related *w*AlbA strain which was also transferred reached high somatic densities [30]. The low fitness costs associated with *w*AlbB and the absence of native *Wolbachia* in *Ae. aegypti* suggests that restrictive host-factor evolution with this symbiont/host combination may be slow, perhaps requiring much longer evolutionary timescales than are relevant in the context of dengue control programmes.

The *w*AlbB strain was successfully established in several sites in urban Kuala Lumpur in 2018, and has maintained high frequencies since [1]. However, the persistence of *w*AlbB in wild *Ae. aegypti* populations is contingent on the maintenance of high rates of maternal transmission and CI given that this strain has some fitness costs [31]. The *w*AlbB maternal transmission rate was complete in the field line, and ovary densities were comparable to those found in the laboratory colony. Maintenance of high ovary density is consistent with evolutionary expectations, as infected females with reduced ovary densities and imperfect maternal transmission would suffer fitness costs in areas of high *Wolbachia* frequency resulting from mating incompatibility with *Wolbachia*-carrying males.

The *w*AlbB field line also displayed maintenance of full CI induction and rescue capacity. Evolutionary models predict that the stability of the CI phenotype may be compromised over time by the evolution and spread of host CI repressors [20,32,33]. Modelling suggests that CI repressors will arise disproportionately in males [20,33], driven by the fitness benefits of *Wolbachia*-carrying male compatibility with *Wolbachia*-free females. The existence of host CI repressors is supported by the lack of strong CI in some native *Wolbachia*/host associations, where the native host becomes fully susceptible to CI upon artificial transfer of a new symbiont strain, or conversely when the native strain induces stronger CI in a non-native host, as observed in several studies in *Drosophila* species [34–36]. Evolutionary models also predict that the symbiont genes underlying the CI phenotype will tend to lose function over time due to a lack of selection on CI levels [21,32]. Consistent with this, the recently identified CI genes often carry loss-of-function mutations [37–40]. However, the persistence of strong CI in many native associations (including in the mosquito species *Ae. albopictus* and *C. quinquefasciatus*), strongly suggests that loss of CI tends to occur over much longer evolutionary timescales, and would, therefore, be highly unlikely to impact on a vector control intervention.

## 4. Conclusion

There is more variability in *Wolbachia* density in wild mosquitoes than in laboratory colonies [1], associated with environmental variability in larval conditions including, for example, exposure to environmental antibiotics that can reduce *Wolbachia* density [41]. Thus, perfect maternal transmission and CI may not be maintained in wild mosquitoes; direct monitoring of field populations for phenotypic stability will be an ongoing component of the *Wolbachia* intervention. The data presented here indicate, however, that *w*AlbB tested in the laboratory has not been attenuated in its invasion, population maintenance and dengue inhibition capacities following its presence for 20 months in a field *Ae. aegypti* population. These data support the sustainability of interventions using *w*AlbB to control dengue transmission. Long-term monitoring across diverse release sites will be required to fully evaluate the potential for *Wolbachia* to reduce the global burden of dengue.

## 5. Methods

### (a) Mosquito strains and rearing

Two *Wolbachia w*AlbB-carrying *Ae. aegypti* lines were used in this study. The first is the original *w*AlbB-carrying line, *w*AlbB.L (Lab), generated as previously described [4] and maintained in controlled laboratory settings for approximately 5 years. A second line, named *w*AlbB.MC, was derived from field-collected eggs from a release area (Mentari Court, 3°04'55.2″N 101°36'39.3″E) in Kuala Lumpur, Malaysia, in December 2019. As previously described [1], releases of *Wolbachia*-carrying mosquitoes for vector control started in this area in October 2017 and ceased in March 2018. Ovitraps (plastic containers, 96 mm height, 67 mm diameter) with 150 ml water and a wooden ovipositor, were placed in apartment buildings in Mentari Court for a week. Eggs from several ovitraps were hatched in the laboratories of the Institute of Medical Research in Kuala Lumpur (KL); the obtained adults (F0) were morphologically identified and *Ae. aegypti* were reared in laboratory settings as previously described [1]. Progeny were sent as eggs to the Centre for Virus Research in Glasgow, UK. Mosquitoes used in this study ranged from F_2_ and F_4_. The wild-type *Ae. aegypti* were colonized from *Wolbachia*-negative mosquitoes collected in control areas of KL in February 2018 and maintained in the insectary of the Centre for Virus Research in Glasgow, UK, for more than 15 generations. *Wolbachia* status was confirmed in wild-type and *w*AlbB-carrying mosquitoes by PCR. Mosquito lines were maintained at standard insectary conditions: 27°C and 70% relative humidity with a 12-h light/dark cycle. Larvae were fed with tropical fish pellets (Tetramin, Tetra, Melle, Germany) and adults maintained with 5% sucrose solution ad libitum. Blood meals were provided by an artificial blood-feeding system (Hemotek, UK) using human blood (Scottish National Blood Transfusion Service, UK). Eggs were collected on a wet filter paper (Grade 1 filter paper, Whatman plc, GE Healthcare, UK), desiccated for 5 days and hatched in deionized water containing 1 g l^−1^ bovine liver powder (MP Biomedicals, Santa Ana, California, USA).

### (b) *Wolbachia* density in the *w*AlbB.MC and *w*AlbB.L lines

The *w*AlbB frequency in the *w*AlbB.MC line was found to be 100% in 48 screened individuals from the F_2_ generation and was measured by quantitative PCR (qPCR) as described below. *Wolbachia* density between *w*AlbB-carrying strains was compared 5 and 10 days PE. Whole-body gDNA was extracted from 24 females from the F_3_ using STE buffer (10 µM Tris HCL pH 8, 100 mm NaCl, 1 mm EDTA) with a 95°C denaturation for 10 min. Additionally, ovaries, salivary glands and midguts (five pools of three organs per replicate) were also dissected from 5-day- and 10-day-old F_4_ females using sterile forceps and needles in a drop of sterile PBS buffer, and immediately transferred in STE buffer for DNA extraction. qPCR analysis was performed using the relative quantification of the *Wolbachia* 16S ribosomal gene (16S_qPCR_F: GAAAGCCTGATCCAGCCATG/16S_qPCR_R: CGGAGTTAGCCAGGACTTCT), against the homothorax gene (HTH) as the reference gene [40]. 2 × SYBR-Green mastermix (Biotool, Houston, Texas, USA) with a BioRad CFX-96 real-time PCR detection system (Bio Rad, Hercules, California, USA) were used for the amplification reaction. The reaction was 95 °C for 5 min, 40× cycles of 95°C for 15 s and 60°C for 30 s, followed by a melt-curve analysis.

### (c) Maternal transmission and cytoplasmic incompatibility

Rates of CI were assessed by performing reciprocal crosses between the wild-type and *w*AlbB.MC lines using 25 virgin females and males in each cross. Mosquitoes were allowed to mate for 3–5 days before blood-feeding. Eggs were desiccated for 5 days at standard insectary conditions (27°C and 70% relative humidity), counted and hatched in water containing 1 g l^−1^ bovine liver powder. Larvae were counted at the L2–L3 stage, and the hatch rate percentage was measured. To assess whether the recaptured line conserved the complete maternal transmission demonstrated in the original line [4], 48 individual progeny resulting from the cross between *w*AlbB.MC females and wild-type males were tested at the larval stage for *Wolbachia* using qPCR as described above.

### (d) Dengue challenges with blood spiked with DENV-2

Susceptibility to DENV-2 dengue virus was assessed using the New Guinea C Strain (Public Health England). The virus was propagated in *Ae. albopictus* C6/36 cells, the supernatant was harvested, concentrated using Amicon Ultra-15 filters (Millipore, IRL) and titrated with fluorescent focus assay (FFA). The primary antibody for DENV was MAB8705 anti-dengue virus complex antibody (Millipore); the secondary antibody was the Goat anti-mouse Alexa Fluor 488, A-11001 (Thermo Scientific, Waltham, Massachusetts, USA). Fluorescent foci were counted by eye (from dilutions with less than 100 foci) and virus titres calculated and expressed as FFU ml^−1^.

Seven-day-old females were offered an infectious bloodmeal consisting of human blood and virus suspension (1.4 × 10^8^ FFU ml^−1^). Engorged females were selected, transferred in containers in a climatic chamber and maintained at 27°C, 70% relative humidity, 12-h light/dark cycle with 5% sugar solution. After 12 days, salivary glands were dissected in sterile PBS and scored for DENV infection using RT-qPCR. RNA was extracted with Trizol (Thermo Fisher) and diluted to 100 ng µl^−1^. cDNA was synthesized with High Capacity cDNA reverse transcription kit (Thermo Fisher). Virus copies were quantified using primers amplifying the viral gene NS5 (NS-5-F: ACAAGTCGAACAACCTGGTCCAT/NS5-R: GCCGCACCATTGGTCTTCTC), and Ct values normalized to the RpS17 mosquito gene (RpS17 F: CACTCCCAGGTCCGTGGTAT/RpS17 R: CACTCCCAGGTCCGTGGTAT).

### (e) Collection of dengue patient blood

Blood from a dengue patient was obtained from the General Hospital Kuala Lumpur, Malaysia. Arrangements were made with the medical officer (MO) in charge of the dengue ward prior to collecting blood. The blood was collected on the same day as feeding. The MO in charge recruited the best candidate based on the pre-set inclusion and exclusion criteria (for information sheet and consent form, see electronic supplementary material, file 1). The patient was initially briefed about the project and the reason for drawing the blood. The patient had complete freedom to give or withdraw consent for the use of their blood. Upon receiving consent, 5 ml of blood was withdrawn and placed in EDTA tubes. Blood was kept on ice at all times and used in feeding experiments on the same day.

### (f) Dengue challenges with patient blood infected with DENV-1

The experimental oral infection was conducted within an arthropod containment level 2 (ACL-2) insectarium. A mouse skin membrane was used in conjunction with a Hemotek Feeding System (Accrington, UK) housed in an isolation glove box. Of dengue patient blood, 1.5 ml was introduced into each feeder. A total of 150 adult female mosquitoes from each group were used in the feeding experiment. Mosquitoes were 3–5 days old and were starved (of sugar solution) overnight. Mosquitoes were allowed to feed for approximately 30 min. The mosquitoes were placed at −20°C for 30 s to allow sorting. Unfed mosquitoes were discarded. Engorged mosquitoes were maintained at 27°C with a relative humidity of 80% (±10%). At each time-point (days 7 and 9), a subset of the living mosquitoes was dissected, with the isolation of midguts and salivary glands. The remaining carcasses were stored. Individual organs and carcasses were kept in tubes containing 100 µl MEM media. Samples were stored at −20°C prior to nucleic acid extraction.

### (g) Nucleic acid extraction and quantitative PCR from patient blood and mosquitoes challenged with DENV-1

Total nucleic acids were extracted from dissected organs using an innuPREP DNA/RNA Mini Kit (Analytik Jena AG, Jena, Germany), enabling the isolation of both RNA and DNA for quantification of DENV and *Wolbachia*, respectively. The DENV strain from patient blood was initially confirmed using real-time reverse transcription PCR (RT-PCR) with DENV 1–4 primer sets [42]. For each run, multiplex reactions were prepared by combining primer/probes for DENV1 + 3 or DENV2 + 4 in a single run. The PCR mixture contained 2 µl of extracted RNA, 5 µl of SensiFAST Probe No-ROX One Step Mix (Bioline, Taunton, MA), 0.4 µl of respective primer combinations (DENV1 + 3 and/or DENV2 + 4) (400 nM), 0.1 µl of the respective probe (400 nM), 0.1 µl reverse transcriptase, 0.2 µl RiboSafe RNase inhibitor and 1.8 µl of nuclease-free water. A RT-PCR was performed using the following programme: 45°C for 10 min, initial denaturation at 95°C for 2 min, followed by 40 cycles with 95°C for 5 s, and 60°C for 10 s. A DENV-1 specific primer set (DENV-1-F: ATCCATGCCCAYCACCAAT/DENV-1-R: GTGGGTTTTGTCCTCCATC/DENV-1-Probe FAM-TCAGTGTGGAATAGGG TTTGGATAG AGGAA-IowaBlackFQ) was used and Ct values were normalized to the RpS17 mosquito gene (RpS17-F: CACTCCCAGGTCCGTGGTAT/RpS17-R: GGACACTTCCGGCACGTAGT/RpS17-Probe: FAM-AGGAGGAAC GTGAGCGCAGAGACA-IowaBlackFQ).

### (h) *Wolbachia* density assessment in DENV-1 infected females

*Wolbachia* density was measured in the extracted DNA from the mosquito carcasses following the dissection of salivary glands and midguts by multiplex qPCR. A mastermix consisting of 2 µl of extracted DNA, 5 µl of PrimeTime Gene Expression Master Mix 2x, 0.375 µl of individual primer sets and 2 µl of nuclease-free water was used. Primers were *Ae. aegypti*-specific (aRpS6-F ATCAAGAAGCGCCGTGTCG/aRpS6-R CAGGTGCAGGATCTTCATGTATTCG/aRpS6-Probe HEX-AGTCCCGCAAGGAAGCCGAA-IowaBlackFQ) and *Wolbachia*-specific (wAlbB1-F CCTTACCTCCTGCACAACAA/wAlbB1-R GGATTGTCCAGTGGCCTTA/wAlbB1-Probe Cy5-TGTTGATCACTTGGCTGTTAGCCCT-IowaBlackFQ). PCRs were performed on a LightCycler 480II system (Roche, Germany).

### (i) Data analysis

All statistical analyses were conducted in the R software v. 3.2.3. Viral titre and *Wolbachia *density data were analysed with linear models after log10 transformation to meet the assumptions of normality and homoscedasticity. *Post hoc* pairwise comparisons were performed with the function glht using a correction for multiple testing (R package multcomp [43]).

## Supplementary Material

Supplementary Figure S1

## Supplementary Material

Information and consent form

## Data accessibility

All raw data have been deposited in the University of Glasgow data repository, link http://dx.doi.org/10.5525/gla.researchdata.1044.
