# Supplementary figures and images for: Wolbachia strain wAlbB maintains high density and dengue inhibition following introduction into a field population of Aedes aegypti
Source: Philos Trans R Soc Lond B Biol Sci. 2020 Dec 28;376(1818):20190809. doi: 10.1098/rstb.2019.0809 (PMC7776933; doi:10.1098/rstb.2019.0809)

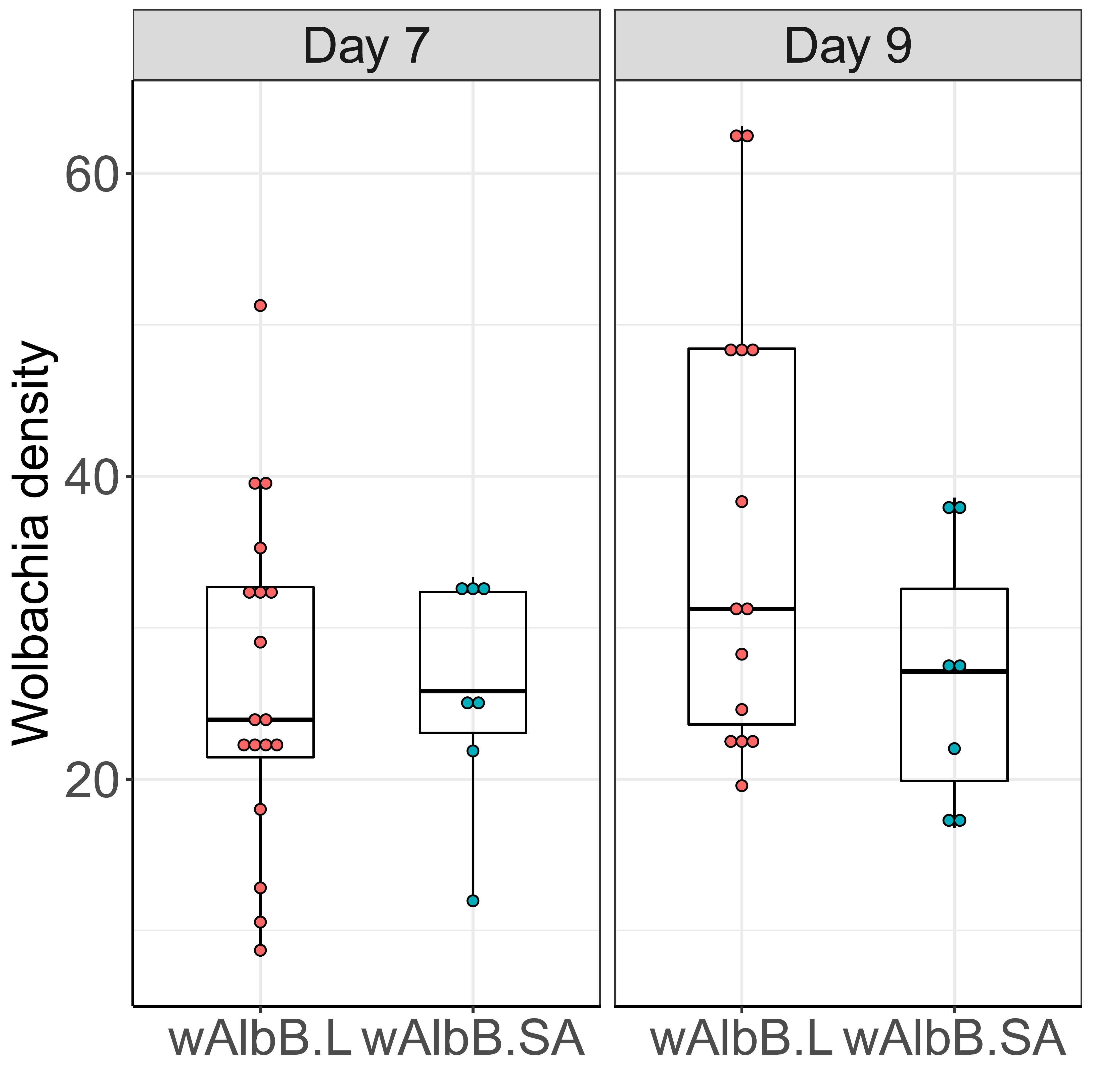

Supplement: Supplementary Figure S1 [file rstb20190809supp1.tiff]
