# Supplementary material for: Wolbachia strain wAlbB maintains high density and dengue inhibition following introduction into a field population of Aedes aegypti
Source: Philos Trans R Soc Lond B Biol Sci. 2020 Dec 28;376(1818):20190809. doi: 10.1098/rstb.2019.0809 (PMC7776933; doi:10.1098/rstb.2019.0809)
Supplement: Information and consent form [file rstb20190809supp2.doc]

**INFORMATION SHEET FOR PATIENT**

**Research Title**

Field evaluation on the impact of *Wolbachia* infected *Aedes aegypti* & *Ae. albopictus* on dengue & chikungunya transmission

**Introduction**

Dengue is the most wide spread viral disease carried by the *Aedes* mosquitoes. To date, there is no specific antiviral treatment, nor a highly effective vaccine. Control of dengue is dependent on suppressing the vector mosquitoes and conventional method of control relies mainly on the use of insecticides such as fogging. Such method, however, has limited effectiveness and new method of dengue control is needed. *Wolbachia* is a microorganism found naturally in many insects including mosquitoes. Laboratory studies indicated that when *Wolbachia* is introduced into *Aedes* mosquitoes, dengue virus cannot grow and so the mosquito cannot transmit the disease to human. *Wolbachia* carrying *Aedes aegypti* were released in a trial sites in Selangor to replace the natural population and hopefully this may stop dengue. To further demonstrate that *Wolbachia* can stop dengue virus from developing in *Aedes aegypti*, we plan to collect the *Wolbachia* carrying *Aedes aegypti* from Selangor and feed them with human patient blood containing dengue virus to determine if dengue virus failed to develop in the mosquito.

**What would this involve?**

At the clinic, your measurement of weight, height, and clinical conditions will be taken. Thereafter, blood will be drawn and about 5ml of venous blood will be drawn. Do inform us if you feel any dizziness/discomfort during and after blood taking, whereby a clinician will immediately attend to you.

Your blood sample will be sent to the Institute for Medical Research and processed before being fed to *Aedes aegypti* that carries the *Wolbachia*. After feeding, the mosquitoes will be maintained for 5-7 days and then tested for the presence of dengue virus.

**QUALIFICATION TO PARTICIPATE**

The doctor in charge of this study or a member of the study staff has discussed with you the requirements for participation in this study. It is important that you are completely truthful with the doctor and staff about your health history. You should not participate in this study if you do not meet all qualifications.

Inclusion criteria:

- 18-50 years old
- Confirmed dengue infection
- Less than 72 hours of fever
- Not having dengue haemorrhagic fever

Exclusion criteria:

- < 18 years old and >50 years old
- Unconfirmed viral infection
- Fever of unknown origin

**The benefits**

There may or may not be any benefits to you. However, information obtained from this study will help the Ministry of Health to improve the control of dengue in future.

**The risks**

As only a small sample of blood is taken from you, there will be negligible risk involved.

**Confidentiality**

Each subject is assigned a number which maintains your confidentiality. All data will be kept in a secure premise by the investigator and will not jeopardize your future treatment. The result of the data obtained will be reported in a collected manner with no reference to a specific individual.

**Do I have to take part?**

The participation into this study is voluntary. If you prefer not to take part, you do not have to give reason and your decision will not affect the treatment given.

**The right to withdraw**

You have the right to withdraw from the study at any time without affecting the future treatment.

**Payment and compensation**

You do not have to pay for participating in this study. Similarly, no payment is available to you for participating in this study.

**If I have any questions, who can I ask at any time point of the study?**

| Dr Norazah Ahmad,  Head,  Infectious Disease Research Centre,  Institute for Medical Research,  Kuala Lumpur.  Tel (office); 03- 2616-2650  Mobile: 019-2281454 | Dr Leong Chee Loon,  Infectious Disease Physician,  Kuala Lumpur Hospital.  Mobile: 013 -2793881 |
| --- | --- |
| Dr Lee Han Lim,  Medical Entomology Unit,  Institute for Medical Reserach, K.L.  Tel (office): 03-2616-2688  Mobile: 016-6238039 | Dr Nazni Wasi Ahmad,  Medical Entomology Unit,  Institute for Medical Reserach, K.L.  Tel (office): 03-2616-2687  Mobil: 012-6899777 |

**CONSENT FORM FOR PATIENT**

**Research Title**

Field evaluation on the impact of *Wolbachia* infected *Aedes aegypti* & *Ae. albopictus* on dengue & chikungunya transmission

I ______________________________________(name), IC no: ___________________, have read the information of this study and have also been given the explanation by the researcher about the

purpose of this document. I understand the aims of the study including its risks and benefits and I have the right to withdraw at any time, without penalty.

I ***agree/disagree** to participate in the study as stated above.

I ***would like to know/don’t want to know** the result of this study (* delete where necessary)

Signature : ____________________________ Date : _________________________

**Witness**

Name :

IC no :

Signature :

Date:

**Researcher**

Name :

IC no :

Signature :

Date:
